# Financial Impact of Medically Integrated Pharmacy Interventions on Oral Oncolytic Prescriptions

**DOI:** 10.1200/OP.22.00022

**Published:** 2022-05-13

**Authors:** Julianne O. Darling, Austin J. Starkey, Josh J. Nubla, Michael J. Reff

**Affiliations:** ^1^ NCODA, Cazenovia, NY

## Abstract

**PURPOSE:**

As the utilization of oral cancer medications rises, it is vital that cancer centers track costs associated with these expensive medications. This research seeks to report the cost interventions associated with medically integrated pharmacies (MIPs) and mail-order pharmacies.

**METHODS:**

Data collection occurred from October 2016 through May 2021. Volunteers input data from their oncology practice into NCODA's Cost Avoidance and Waste Tracker tool, an innovative easy-to use tool that allows practices to document any cost saving interventions or waste occurrences.

**RESULTS:**

The Cost Avoidance and Waste Tracker tool was used by nearly 50 MIPs across the country. Specifically, 26 practices submitted cost avoidance data, and 37 practices tracked waste associated with oral oncolytic therapy. Among the 26 practices, 677 cost avoidance events led to a total cost avoidance of $7,057,053.73 US dollars (USD). The net cost avoidance for the MIP's was $6,510,971.28 USD compared with $546,082.45 USD for the external mail-order pharmacies. Among the 37 practices that reported waste, 768 events were reported, leading to a total drug waste of $11,275,642.16 USD. Of that, $8,935,612.15 USD was attributed waste from external mail-order pharmacies, whereas $2,429,592.01 USD worth of drug waste was reported from MIPs.

**CONCLUSION:**

Medically integrated dispensing of oral oncolytic therapies allows for increased pharmacy oversight, leading to increased cost avoidance and reduced waste for patients and third-party payers. Although these data are difficult to compare because of the complexity of workflows at different dispensing sites, the real-world financial differences between medically integrated dispensing and mail-order pharmacies appear to be significant.

## BACKGROUND

In 2018, 80% of all new drug approvals were oncology medications.^1^ As the approval and prescribing of oral cancer medications continues to rise, it is vital that cancer centers can track additional costs associated with these expensive medications. The average monthly cost of chemotherapy drugs can range from $1,000 US dollars (USD) to $12,000 USD.^2^ This cost varies on the basis of the drug and type of cancer being treated.^2^ The NCODA Cost Avoidance and Waste Tracker (CAWT) is an innovative easy-to-use tool that allows cancer centers to document any cost saving interventions or waste occurrences. The goal of the CAWT is to provide oncology practice sites with an easy to-use tool to track interventions made by the medically integrated team to avoid unnecessary medication cost and to track the waste of oral cancer medications collected within their clinic. Important reasons to track these interventions include showing the value of medically integrated pharmacy (MIP) to third-party payors, tracking pharmacist interventions, and to show the potential benefit of having an integrated pharmacy model for oral oncolytics.

In 2019, NCODA and ASCO assembled an expert panel to complete a systemic review of patient-centered best practices for the delivery of oral oncolytics and supportive care drugs. Following this review, the *Patient-Centered Standards for Medically Integrated Dispensing: ASCO/NCODA Standards* were published in the *Journal of Clinical Oncology*. The best practices for patient-centered care include patient relationships, education, adherence and persistence, safety, refilling of prescriptions, documentation, benefit investigation, medication disposal, and patient satisfaction.^3^ NCODA's CAWT was a recommended resource included in these best practice standards.

For the purpose of this study, MIPs/dispensing (MID) will be defined as a dispensing pharmacy within an oncology center of excellence that promotes a patient-centered, multidisciplinary team approach. The MID is an outcome-based, collaborative, and comprehensive model that involves oncology health care professionals and other stakeholders who focus on the continuity of coordinated quality care and therapies for patients with cancer. MID sites have access to the electronic medical record (EMR) and thus, patients' charts. Since these practice sites have access to the EMR, they can assess when a drug may have been held, reduced, or switched before an oral oncolytic is dispensed. Mail-order pharmacies are defined as offsite pharmacies where prescriptions are mailed to patients without access to any patient health information, often because of insurance requirements.

Several studies show that medically integrated dispensing teams lead to better patient compliance and satisfaction.^4,5^ Frequently, pharmacist interventions play a key role in reducing financial toxicities within the oncology space. One previous study showed that cost savings per intervention was equal to roughly $2,757 USD or $270 USD per prescription.^6^ Data from a single medically integrated site showed an annual estimated net cost avoidance of $1,730,416 USD, and an annual estimated net waste of $119,794 USD for patients who were required to fill through a mail-order pharmacies.^7^ Through the use of NCODA's CAWT tool, this research sought to identify the top oral oncolytics contributing to cost avoidance and waste across the United States, and to quantify the total cost avoidance and waste documented within NCODA's membership over the past 5 years.

## METHODS

Data collection occurred from October 2016 through May 2021. Volunteers from across the country entered data from their oncology practice into NCODA's CAWT tool. NCODA's CAWT is an innovative complimentary electronic database available to all NCODA members and practices. The CAWT tool can track a variety of information in addition to the costs associated with each type of intervention such as the dispensing pharmacy information, type of insurance, payer, pharmacy benefit manager, medication name and strength, expense type, and reasons for the specific expense. The cost of medications is based on the average wholesale price and does not account for insurance coverage, site contracting, copay cards, or patient assistance programs. Pricing is taken from Micromedex Redbook and is calculated to reflect a unit dose cost. Pricing updates and audits are done on a biannual basis by NCODA staff pharmacists to provide the most accurate information. Practices can download their specific CAWT information, but other practices' information is confidential.

Cost avoidance is defined as the amount saved from an intervention that prevents an unnecessary prescription from being filled and sent to the patient. Waste is defined as the amount lost from prescriptions that have been processed and filled for the patient, which are then not used by the patient. Some examples of common interventions in the practice setting include the following:

- If treatment was held and the medication was not dispensed would be a cost avoidance.
- Any therapy change, dose change, patient death, and progression of disease; can be classified as either depending on when in the cycle it occurs. If the same medication (tablet or capsule) can still be used when the dose is changed, it is cost avoidance as you are not having to dispense a whole new prescription. If there is not a way to still use the medication, it is waste.
- When the therapy is changed before starting a new cycle it is cost avoidance; mid cycle is waste.
- Waste also occurs when a mail-order pharmacy sends a refill simply because the refill is due before speaking with the provider. When a mail-order pharmacy fills a prescription multiple days prior (7-9 days) to the patient needing the prescription, so that the prescription arrives near the actual date the patient needs the medication, but a change of therapy occurred during this lead time.

## RESULTS

**FIG 1.**
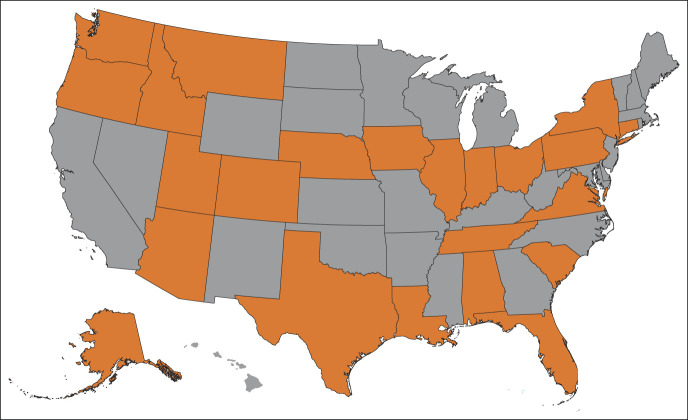
Representation of cost avoidance and waste tracking data collected across the United States. Participating states shown here in orange.

From October 2016 through May 2021, NCODA's CAWT tool was used by nearly 50 MIP practices across the country (Fig 1). Specifically, 26 practices submitted cost avoidance data, and 37 practices used the tool to track waste associated with oral oncolytic therapy (Table 1). Among the 26 practices, 677 cost avoidance events across 60 medications led to a total cost avoidance of $7,057,053.73 USD. The medications leading to the highest amount of avoided costs were palbociclib, lenalidomide, ibrutinib, capecitabine, and regorafenib (Table 2). Practices cited treatment breaks, changes in therapy, dose modifications, and disease progression as the most common types of interventions leading to cost avoidance within their practices.

**TABLE 1.**
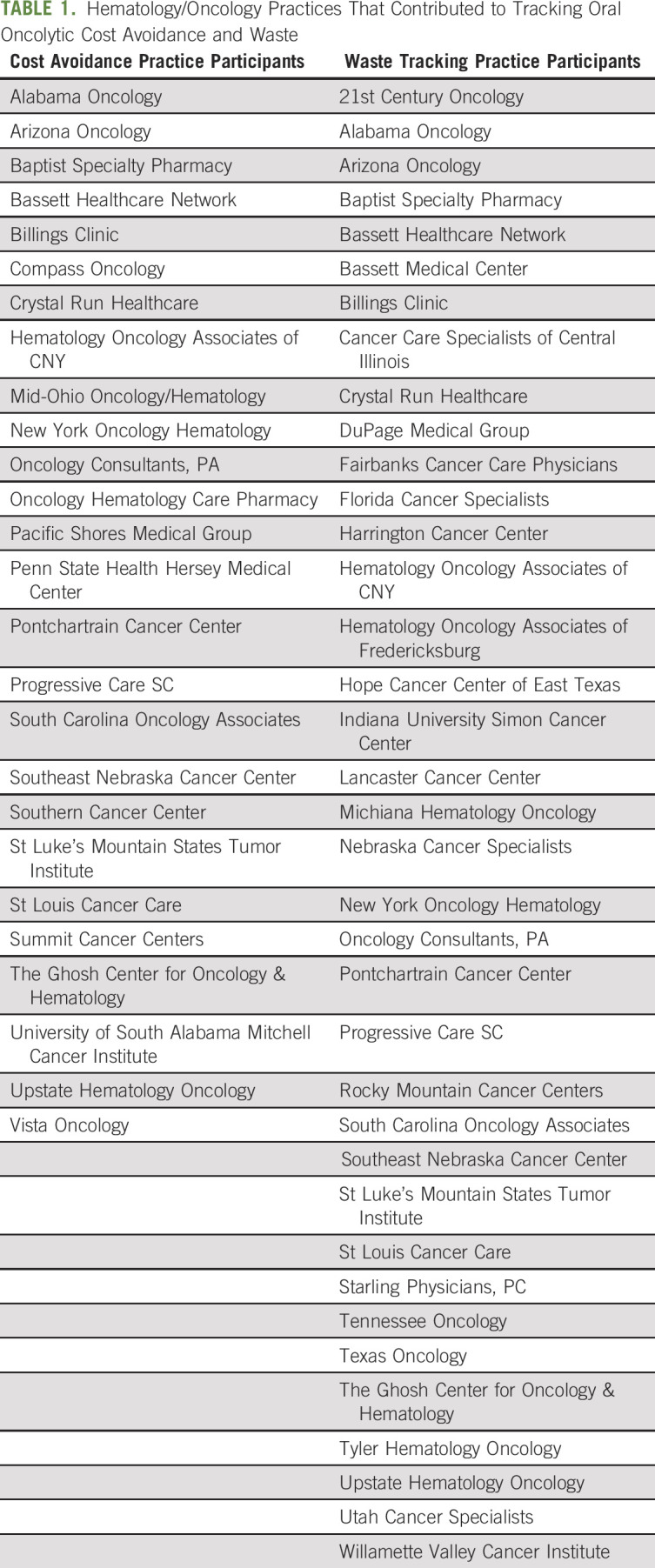
Hematology/Oncology Practices That Contributed to Tracking Oral Oncolytic Cost Avoidance and Waste

**TABLE 2.**
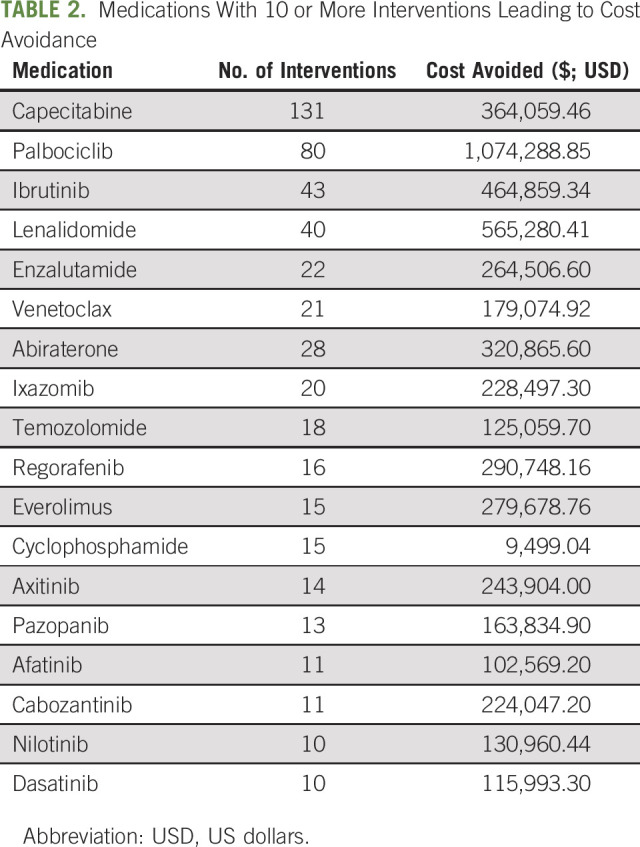
Medications With 10 or More Interventions Leading to Cost Avoidance

Cost avoidance was documented according to dispensing location, allowing the practices to identify interventions for their internal dispensaries compared with interventions for prescriptions sent to external mail-order pharmacies. Of the 677 cost avoidance events, 635 were interventions that occurred for patients filling through the internal MIP, whereas only 40 interventions were documented for scripts filled through external mail-order pharmacies. The net cost avoidance for the MID pharmacies was $6,510,971.28 USD compared with $546,082.45 USD for the external mail-order pharmacies.

Among the 37 practices that reported waste, 768 events were reported across 78 different oral oncolytic medications, leading to a total drug waste of $11,275,642.16 USD. Capecitabine, lenalidomide, palbociclib, everolimus, and enzalutamide had most events leading to waste; however, midostaurin, lenalidomide, everolimus, palbociclib, and cabozantinib had the highest costs associated with their waste events (Table 3). When reported by dispensing location, $8,935,612.15 USD was attributed to waste from external mail-order pharmacies, whereas $2,429,592.01 USD worth of drug waste was reported from MIPs. Some NCODA practice sites are not allowed to accept waste from patients; so, this can limit the amount that can be recorded.

**TABLE 3.**
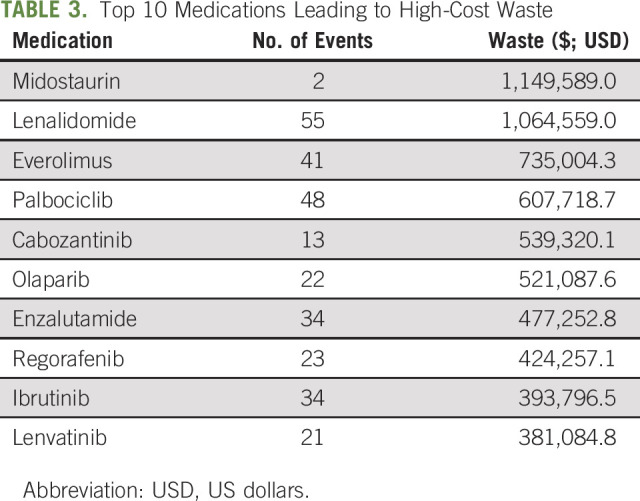
Top 10 Medications Leading to High-Cost Waste

## DISCUSSION

Oral oncolytic workflows and management grow more complex as the number of available oral oncolytic agents continues to climb. A shifting paradigm in oncology practice requires a reconsideration of how oral oncolytic prescriptions are dispensed. Traditional mail-order pharmacies provide education and oral oncolytic refill management, but these services are provided by personnel with limited knowledge of the patient, no access to the patient's EMR, and inadequate access to the patient's oncologist.

Conversely, medically integrated dispensing has demonstrated increased patient and provider satisfaction, clinical benefit to patients, and financial benefit to third-party payers.^7–13^ Over the past 5 years, NCODA members reported a significantly greater amount of cost avoidance in the MID population compared with the mail-order population. Although these data are limited by the fact that practices could choose what to input or omit in the tracker tool, the financial difference between the two models should not be ignored.

MID practices are sometimes limited in the interventions they can make on patients filling externally, leading to less pharmacy oversight and increased waste. The 37 practices that participated in waste tracking over the past 5 years reported significantly more waste from prescriptions filled through mail-order pharmacies compared with those filled through the MID pharmacy. These data are limited by the fact that practices could choose to input certain data and omit other data points. With many practices involved from all over the country, it is unlikely this limitation could explain such a huge difference in waste. This difference is likely a result of MID practices having access to the patient's EMR and accessibility to the providers in the clinic.

Although the data reported in this article display information from close to 50 practice sites all over the United States over a long period of time, there are several limitations to outline regarding this evaluation. As already mentioned, data collection relied on each individual practice site to report cost avoidance and waste for their patients. This implies the MID staff had to manually input all data points into the tracker, which likely means data are under-reported. Additionally, waste was documented only for products that were returned to the clinic, likely leading to much smaller waste calculations. Both the cost avoidance and waste amounts may be much higher than reported. Finally, this evaluation reports all mail-order pharmacies and all MID pharmacies as one. It is a possibility that some individual mail-order pharmacies or MID pharmacies are doing better (or worse) than reflected in this summary. With increased use of the CAWT tool across NCODA's membership, there is the potential to show even more significant differences in cost savings and waste within the MID setting compared with traditional mail-order pharmacies.

In conclusion, medically integrated dispensing of oral oncolytic therapies allows for increased pharmacy oversight, leading to increased cost avoidance and reduced waste for patients and third-party payers. Although these data are difficult to compare because of the complexity of workflows at different dispensing sites, the real-world financial differences between medically integrated dispensing and mail-order specialty pharmacies appear to be significant. The adoption of a standardized waste tracker and cost avoidance tool across the country could provide better insight into the impact of different dispensing models for oral oncolytics.

## Notes

Julianne O. Darling **Honoraria:** Pharmacy Times **Consulting or Advisory Role:** Epizyme **Speakers' Bureau:** Merck, AstraZeneca, Seattle Genetics
